# Antimicrobial effects of *Citrus sinensis* peel extracts against dental caries bacteria: An *in vitro* study

**DOI:** 10.4317/jced.52493

**Published:** 2016-02-01

**Authors:** Sapna B. Shetty, Prabu Mahin-Syed-Ismail, Shaji Varghese, Bibin Thomas-George, Pathinettam Kandathil- Thajuraj, Deepak Baby, Shaista Haleem, Sreeja Sreedhar, Darshan Devang-Divakar

**Affiliations:** 1 Senior Lecturer, Sharavathi Dental College and Hospital, Shimoga, Karnataka, India; 2 Assistant Professor, Department of Conservative Dentistry and Endodontics, IBN Sina National College of Medical Studies, Jeddah, Kingdom of Saudi Arabia; 3 Professor, Department of Orthodontics, P.S.M College of Dental Sciences and Research, Akkikavu, Thrissur, Kerala, India; 4 Associate Professor, Department of Periodontics, IBN Sina National College of Medical Studies, Jeddah, Kingdom of Saudi Arabia; 5 Professor and HOD, Department of Conservative Dentistry and Endodontics, P.S.M College of Dental Sciences and Research, Akkikavu, Thrissur, Kerala, India; 6 Reader, Department of Conservative Dentistry and Endodontics, P.S.M College of Dental Sciences and Research, Akkikavu, Thrissur, Kerala, India; 7 Post Graduate Student, Department of Aesthetic and Restorative Dentistry, University of Manchester, London, UK; 8 Reader, Department of Conservative Dentistry and Endodontics, Sri Shankara Dental College and Hospital, Varkala, Trivandrum, Kerala, India; 9 Dental Biomaterials Research Chair, Dental Health Department, College of Applied Medical Sciences, King Saud University, Riyadh 11433, Kingdom of Saudi Arabia

## Abstract

**Background:**

Ethnomedicine is gaining admiration since years but still there is abundant medicinal flora which is unrevealed through research. The study was conducted to assess the *in vitro* antimicrobial potential and also determine the minimum inhibitory concentration (MIC) of *Citrus sinensis* peel extracts with a view of searching a novel extract as a remedy for dental caries pathogens.

**Material and Methods:**

Aqueous and ethanol (cold and hot) extracts prepared from peel of *Citrus sinensis* were screened for *in vitro* antimicrobial activity against *Streptococcus mutans* and *Lactobacillus acidophilus*, using agar well diffusion method. The lowest concentration of every extract considered as the minimal inhibitory concentration (MIC) values were determined for both test organisms. One way ANOVA with Post Hoc Bonferroni test was applied for statistical analysis. Confidence level and level of significance were set at 95% and 5% respectively.

**Results:**

Dental caries pathogens were inhibited most by hot ethanolic extract of *Citrus sinensis*peel followed by cold ethanolic extract. Aqueous extracts were effective at very high concentrations. Minimum inhibitory concentration of hot and cold ethanolic extracts of *Citrus sinensis* peel ranged between 12-15 mg/ml against both the dental caries pathogens.

**Conclusions:**

*Citrus sinensis*peels extract was found to be effective against dental caries pathogens and contain compounds with therapeutic potential. Nevertheless, clinical trials on the effect of these plants are essential before advocating large-scale therapy.

** Key words:**Agar well diffusion, antimicrobial activity, dental caries, Streptococcus mutans, Lactobacillus acidophilus.

## Introduction

Resistant bacteria represent a challenge in the treatments of various well-known infections and necessitate the need to find new substances with antimicrobial properties to be used against these microorganisms. Plants have anchored to the mother earth long before man can set his feet on earth. Mankind has been gifted with resources for existence much earlier than arrival of life on earth. The World Health Organization (WHO) estimates that about 80% of the population still depends upon herbal medicines for the treatment of various diseases due to easy availability, economic reasons and lesser side effects. Grounds of medical pharmacology have been constructed by herbal remedies for ages and have formed a basis of traditional systems of medicines. Popularity gained by herbal medicines is due to better patient acceptance. Availability of medicinal plants is not a problem especially in developing countries like India, which is having rich agroclimatic, cultural and ethnic biodiversity. India is the largest producer of medicinal herbs and is appropriately called the botanical garden of world (1). Orange, the tasty, juicy fruit, belonging to the family Rutaceae is botanically known as *Citrus sinensis*. *Citrus sinensis* is one of the most important and widely grown fruit crop, with total global production reported to be around 120 million tons. Orange trees are widely cultivated in tropical and subtropical climates for its tasty juice and medicinal value (2).

Many medicinal properties of orange peel extract, such as against colic, upset stomach, cancer, diuretic, cormunative, immuno – enhancing, stomachic, tonic to digestive system, immune system and skin has been listed. It is also used to treat and prevent vitamin deficiencies, colds, flu, and scurvy and helping to fight viral and bacterial infections (3). Antibacterial effects of orange peel have also been demonstrated in the literature. Mehmood *et al.* (2015) showed potent antibacterial activity (against Enteric pathogens) of extract from Orange peels (4). Orange peel extract was also found to be effective against Klebsiella pneumonia by Akdemir (2015) (5).

One of the infectious and major oral health problems affecting mankind since years is dental caries. While *Streptococcus mutans* bacteria is the main cause of tooth decay, *Lactobacilli* characteristically cause existing carious lesions to progress (6). Hence the dental caries pathogens *Streptococcus mutans* and *Lactobacillus acidophilus* were used as test microorganisms for our study.

Thorough literature search did not reveal any studies investigating the effect of orange peel extract on oral disease pathogens.

Hence, the present study was undertaken with the following objectives:

1. To assess and compare the *in vitro* antibacterial properties of different extracts of *Citrus sinensis* against common dental caries pathogens.

2. To determine the minimum inhibitory concentration (MIC) of each extract of both the plants against each pathogen with a view of searching a novel extract as a remedy for dental caries.

## Material and Methods

The study protocol was reviewed and approved by the Institutional Ethical Committee of College of Applied Medical Sciences, King Saud University, Riyadh.

-Procurement of plant material

Oranges (*Citrus sinensis*) were purchased from local market and orange peels were obtained.

-Extraction

The peels were carefully washed under running tap water followed by sterile distilled water. These were air dried at room temperature (30°C) for two days, pulverized to a fine powder using a sterilized mixer grinder and stored in airtight bottles. Two different solvents namely ethanol (hot and cold) and water (hot and cold) were used for extraction to obtain a total of 4 extracts. For the purpose of extraction, a 10 g amount of the pulverized peel was separately soaked in 100 ml of ethanol (96%) and cold sterile distilled water for 24h. Also the same amount (i.e. 10 g) of pulverized peel was immersed in 100ml of hot sterile distilled water (100°C) and allowed to stand for 30 min on a waterbath with occasional shaking and kept undisturbed for 24 h. Each preparation was filtered through a sterilized Whatman No.1 filter paper and the filtered extract was concentrated under vacuum below 40°C using Heidolph, VE-11 rotaevaporator. The dried extract thus obtained was exposed to UV rays for 24h and checked for sterility on nutrient agar plates and stored in labelled sterile bottles in a freezer at 4°C until further use (7).

-Qualitative analysis on phytochemical constituents (8)

Test for tannins

0.5g of powdered sample of each plant was boiled in 20ml of distilled water in a test tube and then filtered. The filtration method used here was the normal method, which includes a conical flask and filter paper. 0.1% FeCl3 is added to the filtered samples and observed for brownish green or a blue black colouration, which shows the presence of tannins.

Test for saponins

2g of powdered samples of each plant was boiled separately with 20ml of distilled water in a water bath and filtered. 10ml of the filtered sample was mixed with 5ml of distilled water in a test tube and shaken vigorously to obtain a stable persistent froth. The frothing was then mixed with 3 drops of olive oil and observed for the formation of emulsion, which indicated the presence of saponins.

Test for flavonoids

A few drops of 1% NH3 solution was added to the aqueous extract of each plant sample in a test tube. A yellow coloration confirms the presence of flavonoid compounds.

Test for terpenoids

5ml of aqueous extract of each plant sample was mixed with 2ml of CHCl3 in a test tube. 3ml of concentrated H2SO4 was carefully added to the mixture to form a layer. An interface forms with a reddish brown coloration if terpenoids constituent is present.

Test for cardiac glycosides

1ml of concentrated H2SO4 was prepared in a test tube. 5ml of aqueous extract from each plant sample is mixed with 2ml of glacial CH3CO2H containing 1 drop of FeCl3. This mixture was carefully added over to 1ml of concentrated H2SO4 already present in the test tube.

Test for Alkaloids

200mg plant material in 10ml methanol, filtered; a 2ml filtrate + 1% HCl + steam, 1ml filtrate + 6 drops of Dragendroff reagent, orange precipitate indicated the presence of respective alkaloids.

Test for carbohydrates

Molisch’s test was used to detect the presence of carbohydrates. One drop of concentrated sulphuric acid was added to about 1g of the herbal extract, and then three drops of 1% α-napthol in 80% ethanol were added to the mixture, without mixing to form an upper phase. Formation of brown or purple ring at the interphase indicated the presence of carbohydrates.

Test for Phenol

To 2-3 ml of aqueous or alcoholic extract few drops of 5% FeCl3 solution was added. Formation of deep blue-black colour indicated the presence of phenols.

-Test Microorganisms

Two dental caries causing bacteria, *Streptococcus mutans* (MTCC*497) and *Lactobacillus acidophilus* (MTCC*447) were procured from Microbial Type Culture Collection, IMTECH, Chandigarh. These microorganisms were subcultured on the specific media, Brain heart infusion agar (*S.mutans*) and MRS agar (*L. acidophilus*) that were attained from HiMedia Laboratory Pvt. Ltd., Bombay, India and incubated aerobically at 37°C. Identification of all strains was confirmed by standard biochemical and staining methods (7).

-Screening for Antimicrobial Activity

The hot and cold aqueous and ethanol extracts of the *Citrus sinensis* were used for the antimicrobial screening using the agar well diffusion method. The media was punched with 7mm diameter wells and were filled with various concentrations of the extracts 5mg/ml, 10mg/ml, 15mg/ml, 20mg/ml and 25mg/ml. The plates were then incubated at 37ºC for 24 hours. After incubation, zone of growth inhibition for each extract was measured in millimeters by using a special scale designed for their purpose by Himedia Laboratories pvt Ltd, measuring diameter between the edges of the lawn. Each extract was tested five times.

-Determination of Minimum Inhibitory Concentration (MIC)

MIC is defined as the lowest concentration of a compound/extract/drug that completely inhibits the growth of the microorganism in 24h. For MIC, 9 dilutions of each extract were done with brain heart infusion (BHI) broth microdilution assay. In the initial tube, 20 microliter of extract was added into the 380 microliter of BHI broth. For dilutions, 200 microliter of BHI broth was added into the next 9 tubes separately. Then from the initial tube, 200 microliter was transferred to the first tube containing 200 microliter of BHI broth. This was considered as 10-1 dilution. From 10-1 diluted tube 200 microliter was transferred to second tube to make 10-2 dilution. The serial dilution was repeated up to 10-9 dilution for each extract. From the maintained stock cultures of required organisms, 5 microliter was taken and added into 2ml of BHI (brain heart infusion) broth. In each serially diluted tube 200 microliter of above culture suspension was added. The tubes were incubated at 37°C for 24 hours and observed for turbidity (7).

-Statistical analysis

The data obtained were analyzed using SPSS (Statistical Package for Social Sciences) version 11.5 (SPSS Inc., Chicago, Illinois, USA). Descriptive statistics (Mean value and SD) along with comparison in mean zone of inhibition between the extracts and at different concentrations of both plants were performed using One way analysis of variance (ANOVA) with bonferroni post hoc. Confidence level and level of significance were set at 95% and 5% respectively.

## Results

**Figure 1:**
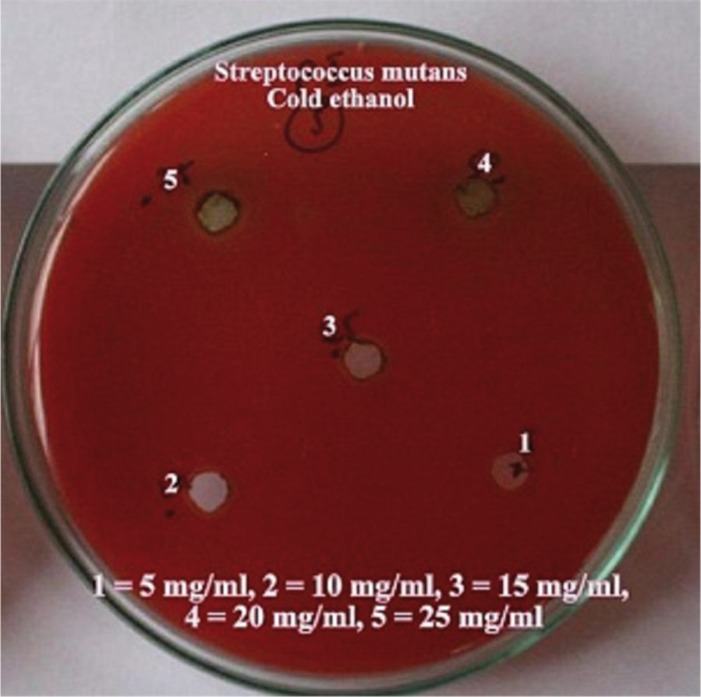
Mean zone of inhibition (mm) of all extracts of *Citrus sinensis* on *Streptococcus mutans*.

**Figure 2:**
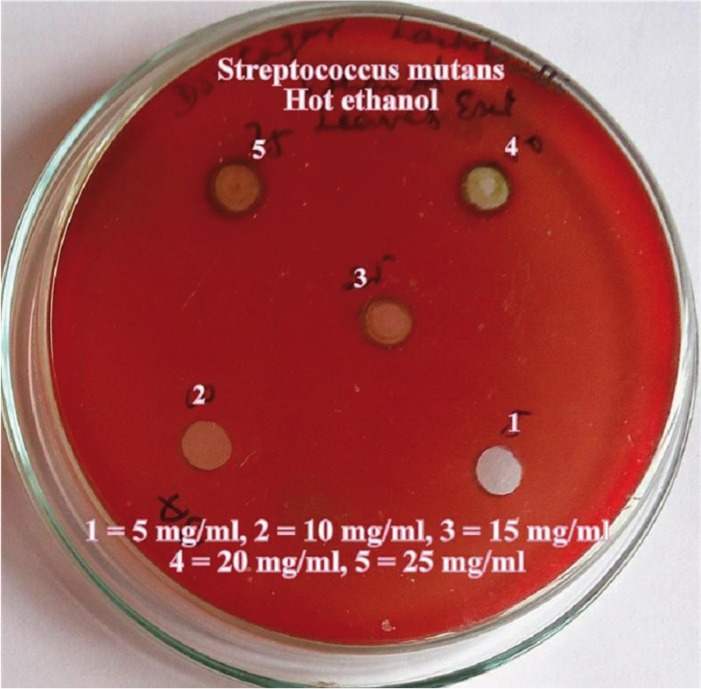
Mean zone of inhibition (mm) of all extracts of *Citrus sinensis* on *Lactobacillus acidophilus*.

**Figure 3:**
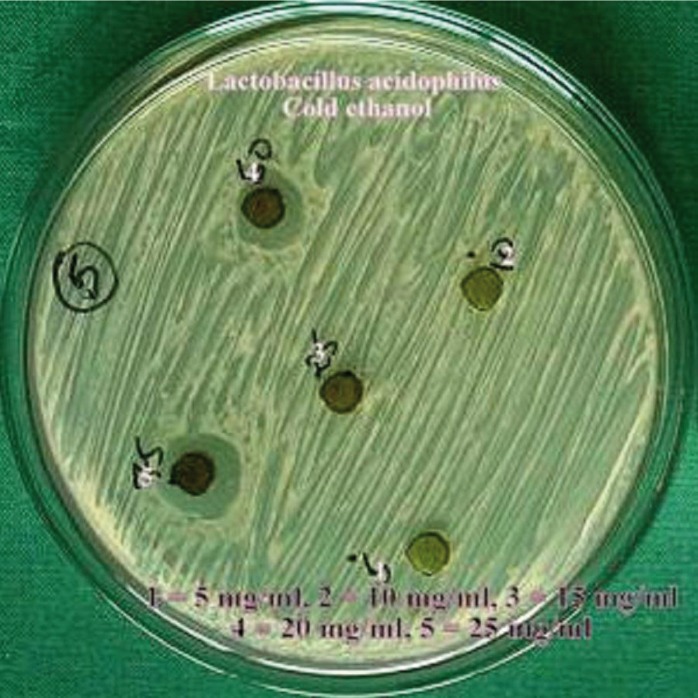
Minimum Inhibitory Concentration (MIC) of *Citrus sinensis* peel extracts against dental caries pathogens on specific media for each microorganism.

**Figure 4:**
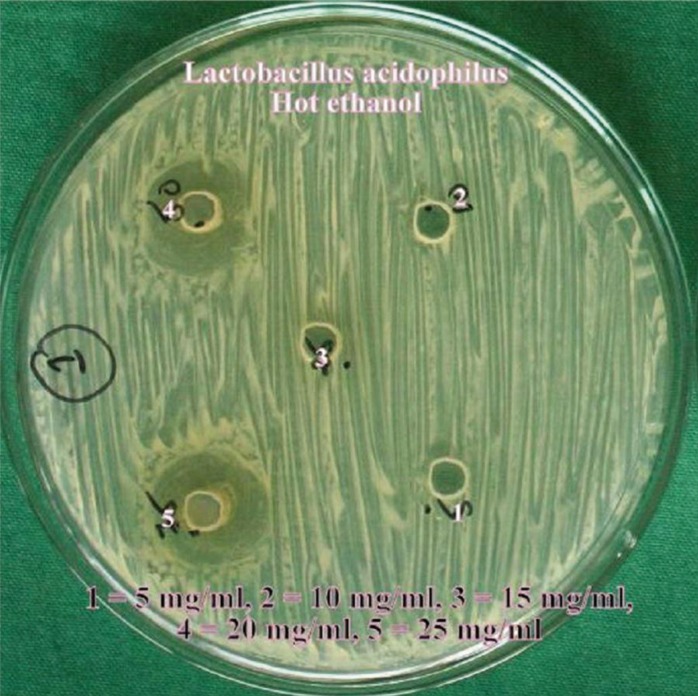
Phytochemical analysis of orange peel extract.

Dental caries pathogens were found to be resistant against hot water and cold water extracts of *Citrus sinensis* (upto 25 mg/ml concentration). Concentration of all extracts was significantly (*p*≤0.05) associated with mean zone of inhibition (in mm). An increase in zone of inhibition was observed with increase in concentration of the extract. Hot ethanolic extract was found to be more effective with greater zone of inhibitions than cold ethanolic extracts against both the dental caries pathogens (Figs. 1-4). At all concentrations, hot ethanolic extracts showed greater zone of inhibition than cold ethanolic extracts against *Streptococcus mutans* and *Lactobacillus acidophilus* ( Table 1, Table 2).

**Table 1:**
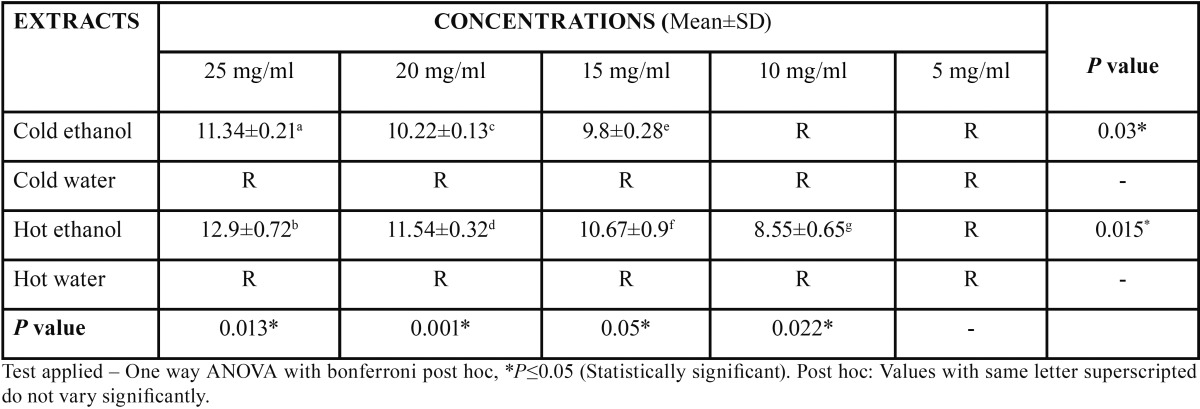
Mean zone of inhibition (mm) of all extracts of *Citrus sinensis* on *Streptococcus mutans*.

**Table 2:**
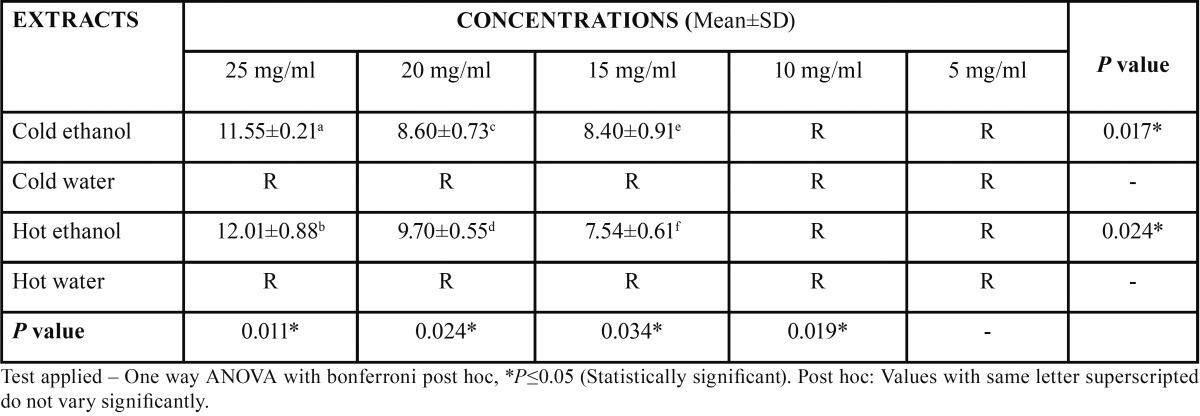
Mean zone of inhibition (mm) of all extracts of *Citrus sinensis* on *Lactobacillus acidophilus*.

Hot and cold water extracts inhibited the microbial growth at very high concentrations of 34.9 and 32 mg/ml against *Sterptococcus mutans*, respectively. Minimum inhibitory concentration of hot and cold ethanolic extracts of *Citrus sinensis* peel ranged between 12-15 mg/ml against both the dental caries pathogens ( Table 3).

**Table 3:**
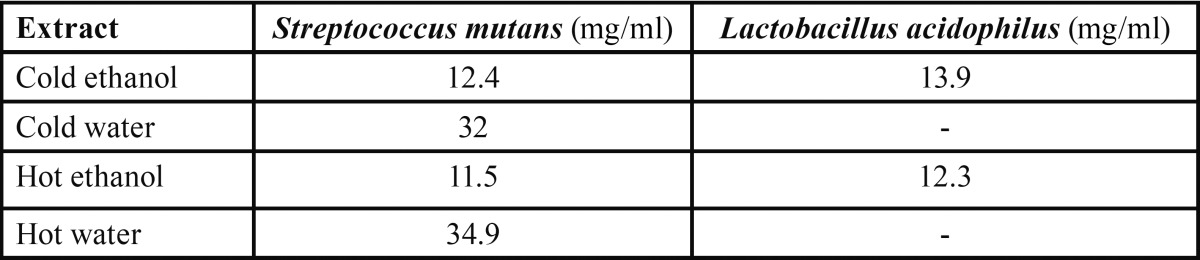
Mean zone of inhibition (mm) of all extracts of *Citrus sinensis* on *Lactobacillus acidophilus*.

Preliminary phytochemical analysis ( Table 4), revealed a slight difference between aqueous and ethanolic extracts. Chemical constituents in aqueous extracts were alkaloids, carbohydrates and glycosides, tannins and phenolics, saponins and flavonoids. Triterpenoids were only present in ethanolic extract.

**Table 4:**
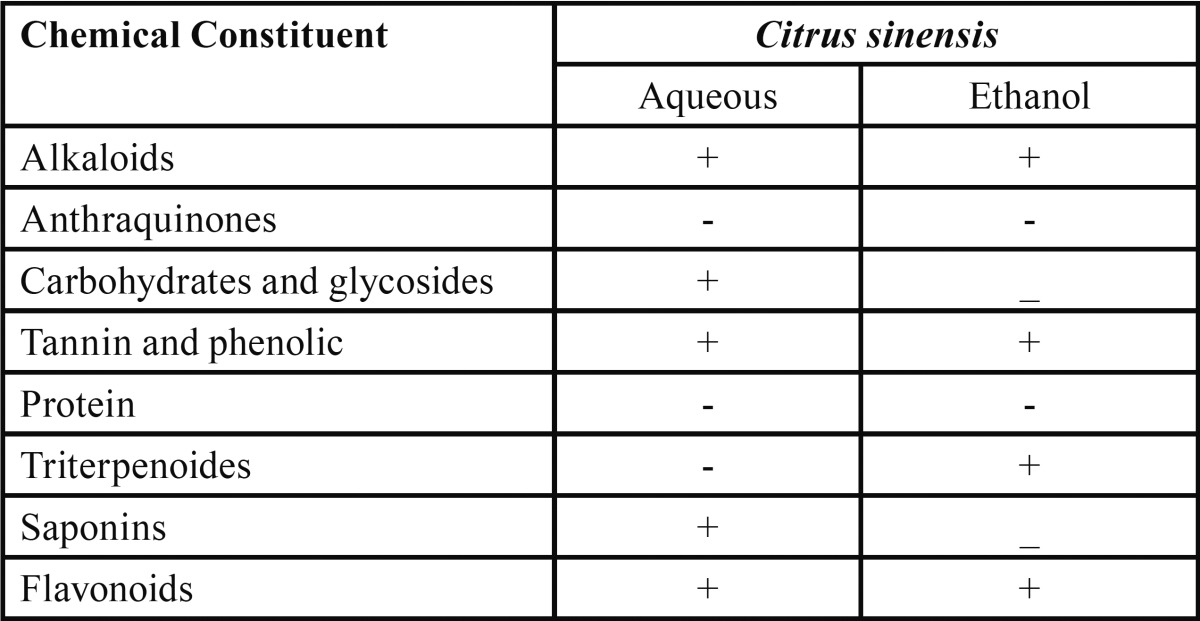
Phytochemical analysis of orange peel extract.

## Discussion

Dental caries is one of the most common chronic infectious diseases in the world. Many herbal remedies have been tried on *in vitro* and *in vivo* against dental caries pathogens and have been found to be effective. Mistry *et al.* (2014) demonstrated the inhibiting effect of Azadirachta indica against dental caries pathogens (9). Hydroalcoholic extracts of fruits like Terminalia chebula also showed potent antibacterial property against *S. mutans* (10). But still no research has been conducted till date assessing the effectiveness of peel extracts of fruits on oral pathogens. Hence the present study assessed the peel of a citrus fruit, *Citrus sinensis* against dental caries pathogens.

Antimicrobial efficacy is usually determined by examining minimum inhibitory concentration, bactericidal effects and other test that commonly utilize various microbial culture techniques. In the present study cultural method employed was agar well diffusion method which offered several advantages such as selective quantification of microorganisms but are laborious and only enumerate bacteria that can grow on agar (11,12).

In the present study, the mean zone of inhibition by orange peel extracts against dental caries pathogens ranged from 8 mm to 13 mm at all concentrations. Pomegranate peel extracts tested against dental caries pathogen, *Streptococcus mutans* showed mean zone of inhibition in the range of 25 mm to 30 mm (13). Naderi *et al.* (2011) tested methanolic extracts of Iranian green tea and black tea against dental caries pathogens. The mean zone of inhibition were found to be 9.5mm and 10.9 mm respectively (14).

Ethanolic extracts showed better antimicrobial activity than aqueous extracts in the present study. Moreover, minimum inhibitory concentrations of the aqueous extracts were much higher than the ethanolic extracts. Cowan (1999) (15) reported that the potency of Citrus fruit peel is enhanced by the type of solvent used indicating that there are some active ingredients in orange peel which have high antimicrobial effect but which would not be released except when orange fruit peel is used in conjunction with a particular solvent. Cowan mentioned that most of the antibiotic compounds already identified in plants are reportedly aromatic or saturated organic molecules which can easily solubilized in organic solvents.

In the present study, hot extracts were found to be more effective than cold extracts. Similar results were obtained in a study conducted by Jeyaseelan and Jashothan (2012) (16) in which leaf extracts of Ricinus communis L were investigated against *Staphylococcus aureus* and *Escherichia coli* and hot ethanolic extract showed better effectiveness. Jeyaseelan and Jashothan (2012) explained that the better activity of hot extracts may be due to the chemical changes caused by the hot treatment, and the resulting biomolecules may be more active than the biomolecules found in the cold extracts (16).

In the present study, the minimum concentrations of ethanolic extracts of *Citrus sinensis* peel that inhibited the growth of dental caries pathogens in the present study ranged between 11.5-12.5 mg/ml. In agreement with previous study (17), present study also demonstrated an increase in zone of inhibition with increase in concentration. The antimicrobial potency of plants is believed to be due to tannins, saponins, phenolic compounds, essential oils and flavonoids. These compounds are known to be biologically active and therefore aid the antimicrobial activities of the plants. These secondary metabolites exert antimicrobial activity through different mechanisms. Tannin as observed in *Citrus cinensis* peel extract have been found to form irreversible complexes with proline rich protein resulting in the inhibition of cell protein synthesis (18).

Another secondary metabolite compound observed in the ethanolic extract was alkaloid. One of the most common biological properties of alkaloids is their toxicity against cells of foreign organisms. These activities have been widely studied for their potential use in the elimination and reduction of human cancer cell lines (19). Just *et al.* (1998) (20) revealed the inhibitory effect of saponins on inflamed cells and is found to be present in the extracts of *Citrus sinensis* peel. Flavonoids, another constituent of both the plants exhibited a wide range of biological activities like antimicrobial, anti-inflammatory, anti-angionic, analgesic, anti-allergic, cytostatic and antioxidant properties (21). Terpenoids observed in ethanolic extracts is speculated to be involved in membrane disruption by the lipophilic compounds (22).

Hence, the peels of fruits of *Citrus sinensis* which are generally treated as wastes can serve as an effective and economical antimicrobial agent as they are available for no cost, have no side effects. In future, *in vivo* clinical studies should be conducted to conform *in vitro* results and for the assessment of safety and efficacy by incorporating these plant extracts into dental products such as mouth rinses and tooth pastes.

## Conclusions

*Citrus sinensis* peels extract demonstrated *in vitro* antimicrobial activity against dental caries pathogens warranting for further *in vivo* clinical studies to determine the exact dosages and its effectiveness in practical situations. Toxicity studies should also be done to determine safety. Need of the hour is to execute more and more screening of natural products or plant parts to set a primary platform for further phytochemical, pharmacological and *in vivo* studies that may open the possibilities of finding new clinically effective antibacterial compounds against dental caries and other bacterial resistant pathogens.
